# Extracellular matrix supports healing of transected rabbit Achilles tendon

**DOI:** 10.1016/j.heliyon.2018.e00781

**Published:** 2018-09-10

**Authors:** Marija Lipar, Boris Zdilar, Mario Kreszinger, Marijana Ćorić, Berislav Radišić, Marko Samardžija, Rado Žic, Marko Pećin

**Affiliations:** a Faculty of Veterinary Medicine, University of Zagreb, Heinzelova 55, 10000 Zagreb, Croatia; b Hospital Sveti Duh, Ulica Sveti Duh 1, 10000 Zagreb, Croatia; c School of Medicine, University of Zagreb, Ulica Šalata 2, 10000 Zagreb, Croatia

**Keywords:** Surgery

## Abstract

Extracellular matrix (ECM) has been intensively used in cardio surgery. The main goal of this research was to determine if Achilles tendon healing could be promoted by applying extracellular matrix scaffold (CorMatrix®, USA). Sixteen (n = 16) New Zealand white mature rabbits (*Oryctolagus cuniculus*) were randomly allocated into two groups. Following complete surgical transection, rabbits in group A (ECM applied) (n = 8) had their Achilles tendons reconstructed using both, nylon suture and extracellular matrix scaffold, whereas in group B (without ECM) the tendons were reconstructed using nylon suture only. After four weeks, the rabbits were euthanized and tendon samples harvested and stained with hematoxylin eosin, Mallory, and Gomory and subsequently histologically analyzed according to modified Bonnar scale. Group B had significantly stronger inflammatory response, including abundant cell infiltration and neovascularization. In group A collagen fibers were predominantly found, whereas in group B reticular fibers were more abundant. Extracellular matrix scaffold has been found to have the real potential for promoting tendon healing through accelerating collagen formation, which is crucial for restoring biomechanical properties of a tendon, decreasing peritendineous adhesion formation, and reducing inflammatory edema and subsequently pain.

## 1 Introduction

Tendons are transducers of contractile force from muscles to bones and in that way they participate in movement. The biggest, thickest and strongest tendon in human, as well as in animal body, is the Achilles tendon [1]. In general, tendons have poor self-regenerating ability and very often it is impossible to achieve full recovery because the scaring tissue has inferior biomechanical properties due to irregular fiber arrangement and forming of peritendineous adhesions, and this is considered to be the additional risk factor for re-injury.

Injuries and subsequent inflammatory response in tendineous tissue are the common cause of pain and movement disorders. The adequate treatment usually includes a long term recovery period [2]. The injured tendon seems to be the most metabolically active during the first six months following surgical reconstruction [3]. In one clinical study, growth factors on porcine collagen scaffold and fibrin scaffolds were used in combination with standard surgical repair to provide mechanical support and promote healing following subcutaneous tendon injuries [4].

In the last decade a series of papers dealing with the improvement of tendon healing were published; the healing has been stimulated with ultrasound waves [5], bone morphogenetic proteins (BMP-2) [6], enzyme collagenase [7], platelet rich plasma (PRP) [8, 9] and mesenchymal cells [10, 11].

CorMatrix^®^ (Cardiovascular, Roswell, GA, USA) is the extracellular matrix in a form of tissue scaffold that originates from porcine intestine and is widely used in cardiovascular surgery. However, biological and biomechanical properties of that material have not yet been sufficiently explored. The predominant component of CorMatrix^®^ is collagen, which is frequently used in tissue engineering due to simplicity of use, remodelling properties, lack of immunogenicity, absorbability, and high potential to promote native tissue growth [12, 13]. CorMatrix^®^ has been the focus of recent clinical studies when it was successfully used in reconstruction of the left coronary artery in a child [14], reconstruction of heart valves in adults [15], reconstruction of the aortic aneurysm [16], reparation of arterio-venous fistula aneurysms [13], closing of laryngo-tracheal-oesophageal clefts [17], and reconstruction of congenital heart defects in infants [18]. In cardio surgery CorMatrix^®^ was successfully used due to lack of calcification, ability to promote cellular and tissue infiltration, reduction in scar inflammatory response and hyperneovascularisation. Taking into account research data found in a number of recent studies that were predominantly published in the field of cardio surgery, the aim of the current study was to determine whether CorMatrix^®^ could be having a positive effect on tendon healing.

## 2 Materials and methods

### 2.1 Human and animal rights

The current experiment comply with the ARRIVE guidelines and it has been carried out in accordance with the U.K. Animals (Scientific Procedures) Act, 1986 and associated guidelines, EU Directive 2010/63/EU for animal experiments, or the National Institutes of Health guide for the care and use of Laboratory animals (NIH Publications No. 8023, revised 1978) and we confirm that such guidelines have been followed in the manuscript.

### 2.2 Ethical statement

Ethical committee at Faculty of Veterinary Medicine, University of Zagreb, Croatia and ethical committee at Ministry of Agriculture, Croatia approved the current study (Class: 640-01/13-17/35, Number: 251/61-01/139-13-1), founding by Ministry of Science, Republic of Croatia.

Sixteen (n = 16) New Zealand white mature rabbits (*Oryctolagus cuniculus*), body weight ranging from 4 to 5 kg were randomly allocated into two groups. Rabbits were sedated with 2-alpha agonist xylazine in combination with dissociative agent ketamine both applied and anaesthesia was maintained with 2% isoflurane applied through the face mask. Immediately after induction of anaesthesia, a single dose of 2% lidocaine was applied epidurally to obtain local analgesia. Surgical area was aseptically prepared. Surgery was performed without magnification tools. Achilles tendons were exposed, clamped with Pean haemostatic forceps and completely transected in their middle part. Tendon reconstruction was done with non-absorbable monofilament nylon (Dermalon^®^ USP 2-0, Upjohn, USA) using Kessler suture pattern. In group A (n = 8) extracellular matrix scaffold (CorMatrix^®^) was attached to tendon with nylon (Dermalon^®^ USP 2-0, Upjohn, USA) using simple interrupted suture pattern. In group B (n = 8) tendons were surgically reconstructed in the same manner, but without using the extracellular matrix scaffold (CorMatrix^®^). In both groups talocrural joint arthrodesis was performed using Kirschner wires and the joints were kept in physiological angle. In both groups paratendon was reconstructed with nylon suture (Dermalon^®^ USP 3-0, Upjohn, USA) using simple running suture pattern, while skin closure was also performed with nylon suture (Dermalon^®^ USP 4-0, Upjon, USA) using simple interrupted suture pattern. In order to minimise the impact that variable surgical technique could be having on the results of this study, the same surgeons performed the surgery in both groups of the animals. Four weeks after surgery, the rabbits were euthanized and tendon samples harvested. Tendon samples were fixed in 4% buffered solution of formaldehyde, embedded in paraffin blocks and sliced in 6 μm thick slides. Histological samples were stained with haematoxylin eosin (HE), Mallory and Gomory. Unlabelled histological samples were presented to the pathologist for obtaining more accurate semi quantitative histological assessment according to modified Bonar scale. Bonar scale score was used over Movie scale for simplifying results and histology assessment of results. Bonar scale has 4 factors for evaluation and Movin eight. Both techniques are commonly used for histologically evaluation of tendon healing. Maffulli 2008 [19] described that Bonar and Movin scale asses the same characteristics of tendon histology. Quantity of collagen and reticulin fibres were graded on histological samples with pluses (+/++/+++); more pluses means more tissue as presented in figures. The presence of inflammatory infiltrate that consisted of lymphocytes, histiocytes, plasma-cells, neutrophils and eosinophils, along with neovascularisation, and forming of collagen and reticular fibres was assessed. Inflammatory infiltrate and neovascularisation were also assessed with pluses (+/++/+++).

### 2.3 Statistical analysis

Statistical analysis was performed by using personal computer and SPSS for Windows XP. In order to compare group A and B descriptive statistical parameters n, standard deviation, arithmetic mean and mode were calculated. The results were statistically analysed by programme STATISTICA. Mann-Whitney U test and T-test were used to set the results and compare groups, statistical significance was set at p ≤ 0.01.

## 3 Results

**Fig. 1:**
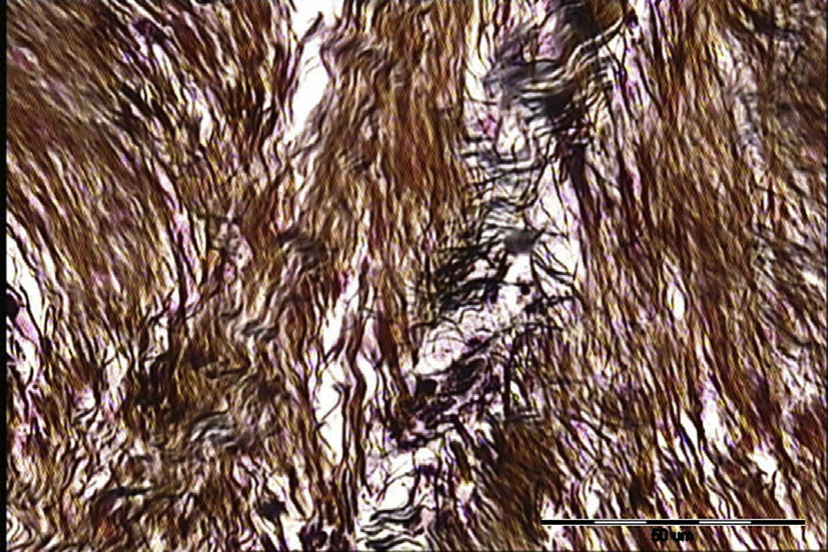
The first grade of reticulin formation during Achilles tendon healing in rabbit model (staining according to Gomory; ×400). Bonar scale validation = +; scale bar = 50 μm.

**Fig. 2:**
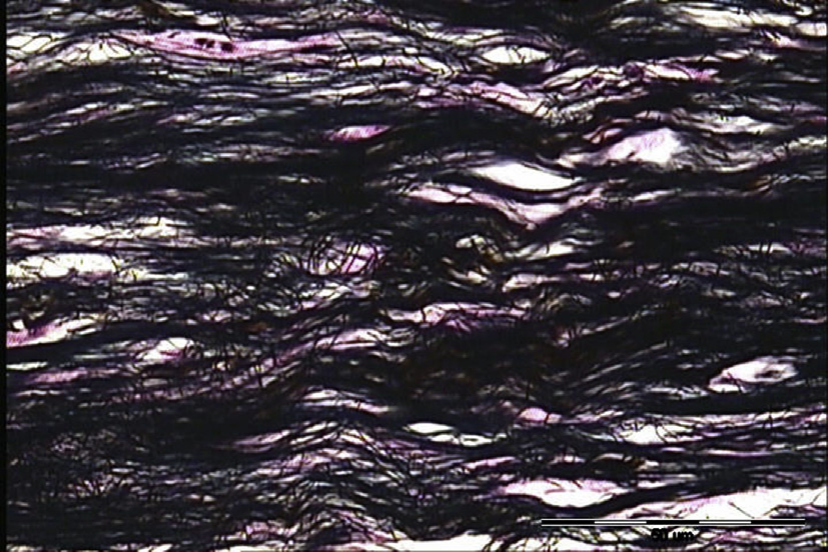
The third grade of reticulin formation doping Achilles tendon healing in rabbit model (staining according to Gomory; ×400). Bonar scale validation = +++; scale bar = 50 μm.

**Fig. 3:**
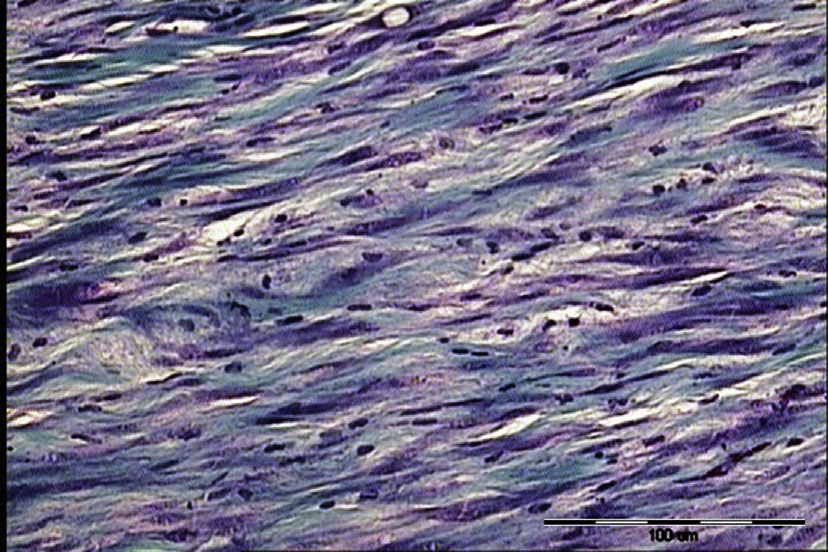
Collagen formation during Achilles tendon healing in rabbit model (staining according to Mallory; ×200). Bonar scale validation = ++; scale bar = 100 μm.

Inflammatory infiltrate contained lymphocyte, hisstiocyte, neutrophil, eosinophil and plasma cells. In A group most frequent value of inflammatory infiltrate was 1 (1.25 ± 0.45) whereas in B group was 3 (2.7 ± 0.67). Significantly more inflammatory infiltrate was in B group (p = 0.00016). Neovascularisation in A group was assessed as 1 (1.42 ± 0.51) in B group as 2 and 3 (2.5 ± 0.53) also, neovascularisation was abundantly significant in B group. p value was 0.00043. Collagen fibers in A group (with ECM applied) were predominately 3 (2.92 ± 0.29) whereas in B group (without ECM) was 2 (1.8 ± 0.42) that is also considered significantly different (p = 0.00002); more collagen fibers were in A group. In contrast statistically significant higher value of reticulin fibers were in B group 3 (2.6 ± 0.52) whereas in A group was 1 (1.25 ± 0.45). P value was 0.00009. In all assessed parameters statistically significant difference between groups was noted. The results are summarised in Table 1. Significant histological differences in fiber quantity are presented in Figs. 1, 2, and 3.

**Table 1:** Presents obtained results of neovascularisation, inflammatory infiltrate, collagen and reticulin fibers assessed histologically according to Bonnar scale in group A and B.

Parameters	Group	Mean value	Standard deviation	Coefficient variation	Mod	P value
Neovascularisation	A	1.42	0.51	0.36	1	0.00043
	B	2.5	0.53	0.21	2 and 3	
Inflammatory infiltrate	A	1.25	0.45	0.36	1	0.00016
	B	2.7	0.67	0.25	3	
Collagen fibers	A	2.92	0.29	0.1	3	0.00002
	B	1.8	0.42	0.23	2	
Reticulin fibers	A	1.25	0.45	0.36	1	0.00009
	B	2.6	0.52	0.2	3

In our study reconstruction and tendon overgrowth were observed in the middle third of the tendon where the natural vascularization is weaker and overgrowth is slower. In the group A extracellular matrix (CorMatrix^®^) had a beneficial effect as it decreased neovascularization and increased inflammatory response.

## 4 Discussion

The main objective of the study was to improve and accelerate Achilles tendon healing in a rabbit model by applying a porcine processed extracellular matrix (CorMatrix^®^). In this study, we used the porcine processed extracellular matrix as a mechanical support to tendon repair, for regeneration of the target tissue without precursors for cell growth, fibrin, antibiotics, platelet-rich plasma and other substances that promote growth.

Formed scar in the tested ligament did not have a fibrous orientation or form as in the intact ligament, indicating that the reparation and regeneration of ligaments is disputable [20]. In the present study, there were twice as many collagen fibres in group A than in group B, indicating that group A was histologically similar to the normal histology of tendon which is well known and extensively studied served as reference control group. Moreover, the reason for such design was in accordance to the RRRR rule. The reasons for such findings may be due to the lesser amount of inflammatory infiltrate and therefore fewer blood capillaries appearing in the overgrowth. The quantity of inflammatory factors was twice as high in group B (without CorMatrix^®^), thereby extending healing time.

Myostatin production has a favourable effect on physically active tendons, it stimulates proliferation and growth and the first stage of overgrowth [21]. In the present study, both groups (A and B) of rabbits underwent tarsal joint arthrodesis to ensure quiescence, to avoid differences in myostatin production as a negative regulator of muscle growth.

A previous study confirmed that morphogenetic protein-2 (BMP-2) in native cartilage enhanced callus formation and provided strength to the cartilage, thereby reducing the amount of reticulin fibres and enhancing collagen formation [6]. In the present study, CorMatrix^®^ acted as an accelerant for rapid collagen fibre formation, therefore reducing local inflammation. Ossification was not detected in rabbit tendons treated with extracellular matrix. This is an undesirable side effect in the chord during assisted overgrowth, as it implies a loss of elasticity of the tendon, which is prone to increased fragility under physiological strain.

*In vitro* cell culture of the flexor tendon of rabbits, chickens, dogs and monkeys was examined, and the thickness of epitenon cell differentiation, cell migration and phagocytosis were evaluated. After 12 weeks, rabbit tendon showed almost complete overgrowth [22]. In the present study, the process of forming epitenon and cell differentiation was faster in group A due to an increase in formed collagen fibres. Cell migration and phagocytosis was also found to be faster in group A, as it had less inflammatory infiltrate as reviewed earlier [23].

PRP is the best stimulator of stem cell migration [1]. However, the results of this study suggest quite the opposite, as the group with stronger neovascularization, and thus more platelets in the lesions, showed significantly fewer collagen fibres. This is due to the more favourable hyperneovascularisation, migration and proliferation of reticulin fibres, which are ultimately the immature fibre form, having with fewer biomechanical properties than collagen fibres.

Alsousou et al. [9] concluded that PRP applied locally promoted and accelerated overgrowth, allowing for collagen type I to be better positioned within the fibre, thereby reducing cellularity and neovascularization, and stimulating glycosaminoglycan production. In the present study, higher neovascularization was observed in group B, suggesting there are more available platelets in the damaged tendon but not accelerated healing, as group A showed more mature fibres, *i.e.* collagen.

Based on the knowledge of the effect of matrix metalloproteinases and the results, it can be concluded that its activity was higher in group A due to the greater quantity of collagen fibres [24]. In a study in which the Achilles tendon was partially resected and left to heal spontaneously, biomechanical testing showed that in the third month postoperative, the tendon submitted only 28.8% of the force in relation to the healthy tendon, followed by 30.2% in the sixth month, and 56.7% in the twelfth month. This is proof that tendons were regenerated and transverse cell connections made between fibres. It takes approximately one year for the fibres of the Achilles tendon to be properly oriented and to achieve over 80% of the strength of healthy tendon [24]. Since we did not directly evaluate the mechanical properties of tendons, but instead performed only histological evaluation, it can be concluded that there were better biomechanical properties owing to more collagen fibres in group A similarly as reported by Axibal and Anderson (2013) [2]. In Group B, reticulin fibres were the dominant type. The shortcomings of this study were that neither biomechanical testing nor blood biomarkers of neovascularization were measured. Also histological assessment of tendons was omitted at multiple time intervals. Long term studies are needed to provide better understanding of CorMatrix^®^ influence in animals and humans tendon healing.

In 55 patients with a painful Achilles tendon, the initial review of neovascularization was present only in 30 tendons and neovascularization spontaneously disappeared within 3 months. Inhomogeneity was found in 35 of 55 tendons [25]. A similar argument is supported by other authors [26]. Therefore, as one of the changes in tendon overgrowth, hyperneovacularization should not be neglected. Conclusions and proposed therapy plans should not be made only on the basis of the scope of neovascularization. The homogeneity of tendons, or the ratio of reticulin and collagen fibres should also be considered, as this is a relevant indicator found in neovascularization occurring in a thinned section of the tendon in 97.3% of cases [27, 28].

Hyperneovascularisation in the tendon healing is an indicator of the success of surgical treatment. No differences were found in collagen quality between the operated and non-operated group according to immunohystochemical analysis [7]. In the present study, significant differences in neovascularization were found between groups. Neovascularization was 50% higher in group B (without CorMatrix^®^).

An earlier study stated that it could not be determined with certainty as to whether neovascularization is necessarily associated with oedema and pain, and that the final outcome cannot be determined based solely on the intensity of neovascularization in the area of the Achilles tendon [26].

In the present study, neovascularization was observed in the context of the incidence of oedema and the inflammatory infiltrate. In group A, which recorded a significantly lower percentage of neovascularization, there was also less inflammatory infiltrate. It could be assumed that tendons in group A were less painful and that the regenerative properties at the end of the experiment in week 8 were significantly better, with histological examination showing a greater amount of collagen fibres. In group B, intensive neovascularization and inflammatory infiltrates were recorded. More immature fibres, *i.e.* reticulin fibres, were also found in the regeneration phase. In the early stage of overgrowth of tendons, those reticulin fibres were gradually and partially replaced by collagen fibres. Therefore, in group B, overgrowth of the tendon was significantly slower, as reticulin fibres were in a significantly higher percentage than in group A. It is assumed that the intensive inflammatory infiltrate and hyperneovascularization slowed the process of tendon overgrowth. A defect in the tendon was found in the middle third, as this is the site of the physiologically minimal vascularization of the tendon. Our research may exclude the beneficial effects of blood clot on tendon overgrowth.

In an earlier study, mesenchymal cells impregnated with cytokines reduced inflammation in tendon healing and increased the concentration of procollagen type I [10, 11]. Human mesenchymal cells stimulate cellular organization and accelerate the maturation of the matrix in the injured tendon [10, 11]. A similar result was obtained in the present study with CorMatrix^®^, but without cell supplements. It is therefore easier to apply and the body is less compromised in terms of an immune response to agents. In all three cases, rapid formation of collagen was noticed, which is essential for tendon functioning.

The study results confirmed the hypothesis that the group of tendons reconstructed with a Kessler suture and with the extracellular matrix set (CorMatrix^®^) (group A) had less inflammatory infiltrate and blood vessels with more collagen fibres. The second group (B) without the use of CorMatrix^®^ had more inflammatory infiltrate, blood vessels and immature collagen fibres.

## 5 Conclusion

In the current research extracellular matrix accelerated collagen formation which is the essential compartment of tendon responsible for biomechanical properties. Furthermore reduced peritendinous adhesion and subsequently reduced edema and pain as a result of very easy application in injured tendon.

## Declarations

### Author contribution statement

Marija Lipar, Marko Samardžija: Conceived and designed the experiments; Analyzed and interpreted the data; Contributed reagents, materials, analysis tools or data; Wrote the paper.

Boris Zdilar, Mario Kreszinger, Marko Pećin: Conceived and designed the experiments; Performed the experiments; Contributed reagents, materials, analysis tools or data; Wrote the paper.

Marijana Ćorić, Berislav Radišić: Conceived and designed the experiments; Performed the experiments; Analyzed and interpreted the data; Contributed reagents, materials, analysis tools or data; Wrote the paper.

Rado Žic: Conceived and designed the experiments; Contributed reagents, materials, analysis tools or data; Wrote the paper.

### Funding statement

This research did not receive any specific grant from funding agencies in the public, commercial, or not-for-profit sectors.

### Competing interest statement

The authors declare no conflict of interest.

### Additional information

No additional information is available for this paper.
